# Spectrophotometric Evaluation of Polyetheretherketone (PEEK) as a Core Material and a Comparison with Gold Standard Core Materials

**DOI:** 10.3390/ma9060491

**Published:** 2016-06-20

**Authors:** Bogna Stawarczyk, Philipp Schmid, Malgorzata Roos, Marlis Eichberger, Patrick R. Schmidlin

**Affiliations:** 1 Department of Prosthodontics, Dental School, Ludwig-Maximilians-University Munich, Goethestrasse 70, Munich 80336, Germany; marlis.eichberger@med.uni-muenchen.de; 2 Clinic of Preventive Dentistry, Periodontology and Cariology, Center of Dental Medicine, University of Zurich, Plattenstrasse 11, Zurich 8032, Switzerland; philipp.schmid@swissdentalcenter.ch (P.S.); Patrick.Schmidlin@zzm.uzh.ch (P.R.S.); 3 Division of Biostatistics, Epidemiology Biostatistics and Prevention Institute, University of Zurich, Hirschengraben 84, Zurich 8001, Switzerland; mroos@ifspm.uzh.ch

**Keywords:** polyetheretherketone (PEEK), color, spectrophotometer, chair-side color measurements

## Abstract

This study investigated the colorimetric properties of different veneering materials on core materials. Standardized specimens (10 mm × 10 mm × 1.5 mm) reflecting four core (polyetheretherketone (PEEK), zirconia (ZrO_2_), cobalt–chromium–molybdenum alloy (CoCrMo), and titanium oxide (TiO_2_); thickness: 1.5 mm) and veneering materials (VITA Mark II, IPS e.max CAD, LAVA Ultimate and VITA Enamic, all in shade A3; thickness: 0.5, 1.0, 1.5 and 2 mm, respectively) were fabricated. Specimens were superimposed to assemblies, and the color was determined with a spectrophotometer (CieLab-System) or a chair-side color measurement device (VITA EasyShade), respectively. Data were analyzed using three-, two-, and one-way ANOVA, a Chi^2^-test, and a Wilson approach (*p* < 0.05). The measurements with EasyShade showed A2 for VITA Mark II, A3.5 for VITA Enamic, B2 for LAVA Ultimate, and B3 for IPS e.max CAD. LabE-values showed significant differences between the tested veneering materials (*p* < 0.001). CieLab-System and VITA EasyShade parameters of the different assemblies showed a significant impact of core (*p* < 0.001), veneering material (*p* < 0.001), and thickness of the veneering material (*p* < 0.001). PEEK as core material showed comparable outcomes as compared to ZrO_2_ and CoCrMo, with respect to CieLab-System parameters for each veneering material. The relative frequency of the measured VITA EasyShade parameters regarding PEEK cores also showed comparable results as compared to the gold standard CoCrMo, regardless of the veneering material used.

## 1. Introduction

Restoring and replacing teeth with computer-aided design and computer-aided manufacturing (CAD/CAM) [1] has gained in popularity and become a key competence in dentistry. However, oral rehabilitation is delicate in terms of functional and esthetic outcomes and only an adequate material choice and processing can ensure long-term stability and patient satisfaction on teeth and implants [2,3]. Whereas, at first sight, the esthetic appearance of any restoration is of great subjective importance for patient and oral care provider, other significant aspects like biocompatibility, function, and longevity play a substantial role [4].

Whereas veneering materials aim to rebuild the outer body of the tooth, abutment and core materials are required to reinforce the integrity and stability of the restoration [5]. However, the color of the latter may greatly influence the appearance of the whole restoration and may hamper adequate esthetics [6]. Therefore, besides physical-chemical testing, the materials ability to mimic the natural tooth substance with regard to translucency, opalescence, and overall color is also important when screening and evaluating potential restorative materials and combinations thereof [7].

Polyetheretherketone (PEEK) represents a relatively new material and is regarded as a promising alternative in fixed and removable prosthetic dentistry. It is a linear, aromatic, semi-crystalline thermoplastic polymer with notable mechanical properties [8]. Recent studies have shown that it fulfills the basic requirements to be used in the restorative field as it shows adequate mechanical stability and also allows for bonding to conventional veneering materials [9,10]. However, this material may significantly interfere with the aforementioned desired esthetic outcomes, as the clinical use of PEEK as full-coverage monolithic restorations may be notably limited by its low translucency and a grayish or even snow-white color. Therefore, additional resin composite or ceramic materials for veneering are still necessary, especially in the esthetic zone. To date, according to the authors' knowledge, no studies are available, which have investigated color characteristics and optical properties of PEEK as compared to other currently used abutment materials in combination with veneering materials.

Since spectrophotometric technologies (e.g., CieLab-System) are widely used in dental color studies [11], the present study was designed using this technology to assess differences in optical measurements of PEEK as a base material as compared to three frequently used base materials, namely, a metal alloy (so-called gold standard), zirconia, and titanium, when layered with four different veneering materials of different thicknesses. The following six null hypotheses were formulated:

(1) There is no difference in the CieLab-System parameters of assemblies and the modification of the CieLab-System parameters for each veneering material separately.
(2) The veneering materials have no impact on CieLab-System parameters.
(3) The core materials within given assemblies have no impact on the CieLab-System parameters.
(4) The core material has no impact on the modification of the CieLab-System parameters between assembly and veneering material.
(5) The veneering materials have no impact on the VITA EasyShade parameters.
(6) The core materials have no impact on the VITA EasyShade parameters.

## 2. Materials and Methods

### 2.1. Specimen Preparation

In order to test the variety of restorative combinations, different core and veneering materials were evaluated. For this purpose, ten test specimens reflecting four different core materials (PEEK, zirconia (ZrO_2_), cobalt–chromium–molybdenum alloy (CoCrMo), and titanium oxide (TiO_2_)) were prepared in standardized dimensions of 10 mm × 10 mm × 1.5 mm. The materials used in this study are presented in Table 1.

The respective CAD/CAM materials, *i.e.*, PEEK, ZrO_2_, and TiO2, as well as the residue-combustible acrylic VITA CAD-Wax (VITA Zahnfabrik, Lot. No: 22890) for the CoCrMo specimens, were cut into 2-mm-thick slices. ZrO_2_ specimens were sintered (LHT 02/16, Nabertherm GmbH, Lilienthal/Bremen, Germany) according to the manufacturer instructions at a heating rate of 10 °C/min to 1500 °C with a holding time of 120 min. The wax specimens were invested (TeleVest, Siladent, Goslar, Germany; Lot. No: 1304672/12289) and cast in an induction vacuum casting machine (GLOBUCAST, Obodent, Bohmte, Germany) according to the manufacturer‘s instructions using a CoCrMo alloy (Table 1). After cooling, the investment material was removed in an air-abrasion unit (CEMAT NT4, Wassermann, Hamburg, Germany) using 50 µm of Al_2_O_3_ (Cobra, Renfert, Hilzinger, Germany) at a pressure of 2 bar.

In addition, the following CAD/CAM materials—all in A3 chairside color as delivered by the manufacturer—were chosen to simulate the veneering situation: VITA Mark II, IPS e.max CAD, LAVA Ultimate, and VITA Enamic (Table 1). Blanks were cut using a low-speed diamond saw (Well 3241, Well Diamantdrahtsägen, Mannheim, Germany) in accordance with standardized thicknesses of 0.5 ± 0.01, 1.0 ± 0.01, 1.5 ± 0.01, and 2 ± 0.01 mm, respectively. Thereafter, core and veneering materials were polished on both sides under constant water-cooling up to silicon carbide paper (SIC) P4000 (Tegramin-20, Struers, Ballerup, Denmark).

### 2.2. Specimen Assembly and Color Determination

In a first step, the color of the different core materials was determined according to the CieLab-System with a spectrophotometer (CM 3500d, Minolta AG, Dietikon, Switzerland). The measurement tip of the spectrophotometer was always directed towards the middle of the specimens, and the spectrophotometer recorded the *L* (luminosity), *a* (red-green axis), and *b*-values (yellow-blue axis). Additionally, *E*-values were calculated according to the formula: *E* = [(*L*)^2^ + (*a*)^2^ + (*b*)^2^]^1/2^. After the 10 measurements, the means of each material group were determined, and the sample best fitting to this mean value served as the master base material for the measurements to follow. Afterwards, the veneering materials of different thicknesses were superimposed individually, and the measurements were made in triplicates using the spectrophotometer at the veneering thicknesses of 0.5, 1.0, 1.5, and 2.0 mm.

The 2-mm-thick veneering specimens of each material specimen served as a control reference for the color measurements of the CieLab-System measurements for the difference determination (∆*L*, ∆*a*, and ∆*b*) between the different readings of the combined base/veneering material assemblies. The overall color difference was again calculated as ∆*E* = [(∆*L*)^2^ + (∆*a*)^2^ + (∆*b*)^2^]^1/2^, where ∆*L* = *L*(veneering) − *L*(assembly); ∆*a* = *a*(veneering) − *a*(assembly); ∆*b* = *b*(veneering) − *b*(assembly).

The following color definitions for the respective positive (+) and negative (−) values were used for all interpretations:

∆*L* = (+) white, (−) black; ∆*b* = (+) yellow (−) blue; ∆*a* = (+) red, (−) green

In addition to that, a chair-side color measurement device was used to determine the VITA EasyShade of each assembly (VITA EasyShade Compact, Vident, Model # DEASYCBU, Serial Number 20365). Before each measurement, the device was calibrated using the calibration apparatus according to the manufacturer’s instructions. The measurements were repeated three times and coded in an excel sheet.

### 2.3. Statistical Evaluation

Descriptive statistics such as mean and standard deviation were computed. The differences between the groups with respect to CieLab-System parameters and factors such as framework material, veneering material, and veneering material thickness as well as their interactions were determined using three-way ANOVA. The interaction between all factors affected the results. Therefore, the fixed effects of framework material, veneering material, and veneering thickness cannot be compared directly as the higher-order interactions were found to be significant. Consequently, several different analyses using two- and one-way were computed and divided by the level of framework material, veneering material, and veneering depending on the hypothesis of interest. The association between VITA EasyShade values (chair-side tooth shade) and veneering material as well as core material was investigated with a Chi^2^-test. In addition, the relative frequencies of VITA EasyShade values were given using the Wilson approach (SPSS V20, SPSS INC, Chicago, IL, USA). All results for statistical analyses with p-values below *p* = 0.05 were considered to be statistically significant.

## 3. Results

### 3.1. The Evaluation of the Color Properties of the Veneering Materials

The chair side tooth shade was defined with A3 according to the manufacturer of all four veneering materials (Table 1). The measurements in this study showed that VITA Mark II is A2, VITA Enamic A3.5, LAVA Ultimate B2, and IPS e.max CAD B3 (Table 2).

The corresponding color space values (LabE) to the chair-side color are presented in Table 2. LabE-values showed significantly differences between the tested veneering materials (*p* < 0.001). With regard to lightness (L), VITA Mark II (A2) and IPS e.max CAD (B3) showed significantly higher values than VITA Enamic (A3.5) and LAVA Ultimate (B2) (*p* < 0.001). The lowest color opponents dimension was observed for LAVA Ultimate (B2), followed by IPS e.max CAD (B3), VITA Mark II (A2), and VITA Enamic (A3.5), respectively (*p* < 0.001). Among b-color values, VITA Enamic (A3.5) showed the highest b color opponents dimension, followed by VITA Mark II (A2) and LAVA Ultimate (B3). However, the latter were in the same range (*p* = 0.08). Within the global values (*E*), the lowest *E*-values were observed for LAVA Ultimate (B2), followed by VITA Enamic (A3.5) (*p* < 0.001). The highest *E*-values showed VITA Mark II (A2) and IPS e.max CAD (B3), which were not significantly different from each other (*p* = 0.941) (Table 2).

The CieLab-System parameters and VITA EasyShade parameters of the different assemblies between veneering and core material were evaluated.

The three-way ANOVA indicated that the core (*p* < 0.001) and veneering material (*p* < 0.001), and the thickness of the veneering material (*p* < 0.001) had a significant impact on the CieLab-System parameters.

**Figure 1:**
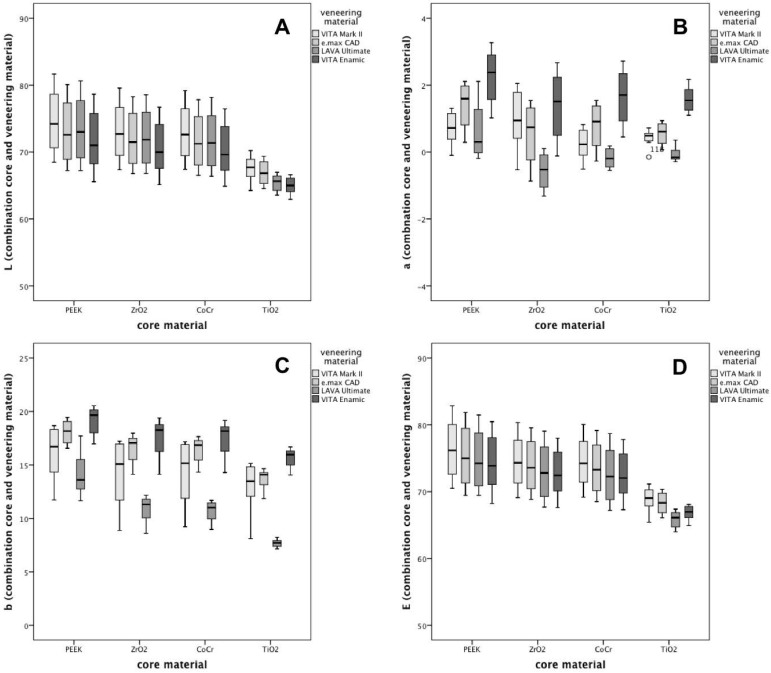
CieLab-System parameters of all tested material assemblies, regardless of the veneering thickness.

In general, TiO_2_ showed significantly lower *L*- and *E*-value as compared to the other core materials (*p* < 0.001; Figure 1). In contrast, with consideration of the *a*-value, PEEK resulted in significantly higher values than the other three core materials (*p* < 0.001). With regard to the *b*-value, PEEK showed again the highest results, followed by ZrO_2_ and CoCr. The lowest *b*-values were observed for TiO_2_. These results were independent of the used veneering material.

With regard to lightness (*L*)-values, VITA Enamic showed the lowest values followed by LAVA Ultimate, IPS e.max CAD and VITA Mark II, respectively (*p* < 0.001). Focusing on *a*- and *b*-values, the same range and order was observed. Lava Ultimate presented the lowest values followed by VITA Mark II, IPS e.max CAD, and VITA Enamic. When considering the global *E*-value, VITA Enamic and LAVA Ultimate were in the same range, whereas IPS e.max CAD and VITA Mark II presented significantly higher *E*-values.

The measured VITA EasyShade parameters showed an impact of the core and veneering material combination (Chi^2^: *p* < 0.001). Core material in combination with VITA Mark II (A2) showed the following predominant tooth shades: A1 (39.4%), A2 (36.3%), respectively (Table 3). For VITA Enamic (A3.5) core, the predominant shades measured were B3 (53.8%) and A3.5 (13.1%). For LAVA Ultimate (B2), tooth shades A1 (36.9%), B2 (31.9%), and A2 (18.8%) were predominant. In contrast, IPS e.max CAD (B3) showed mainly B3 (46.3%) and B2 (20.6%) values. Although all veneering materials were delivered from the manufacturers with A3, this tooth shade was only measured in 11.6% of all combinations in this study.

The relative frequency of the measured VITA EasyShade parameters for PEEK core material (A1: 25%, A2: 17%, B3: 31%) showed comparable results with CoCrMo (A1: 25%, A2: 16%, B3: 31%), regardless of the veneering material used.

### 3.2. Influencing the Overall CieLab-System Parameters through the Core Material

The three-way ANOVA indicated that core (*p* < 0.001) and veneering material (*p* < 0.001) as well as of the thickness of the veneering material (*p* < 0.001) had a significant impact on values ∆*L*, ∆*a*, ∆*b* and ∆*E*. In general, the lightness parameter ∆*L* produced significantly higher values for TiO_2_ as compared with the other core materials, regardless of the veneering material (*p* < 0.001; Table 4). For veneering materials VITA Enamic, LAVA Ultimate, and IPS e.max CAD, comparable influences of the core material were shown, *i.e.*, the lowest ∆*a*-values were recorded in groups combined with PEEK core and then with CoCrMo and TiO_2_ (*p* < 0.001). However, the highest ∆*a*-values were shown in combination with ZrO_2_ (*p* < 0.001). Within groups veneering with VITA Mark II, the combination with ZrO_2_ showed the lowest ∆*a*-values, followed by PEEK and TiO_2_ as well as CoCrMo, respectively (*p* < 0.001). For ∆*b* results, the combination with the core material PEEK revealed the lowest values, followed by ZrO_2_, CoCrMo, and TiO_2_ (*p* < 0.001), regardless of the veneering material. ∆*E*-values revealed a significant influence of the core material regardless of the veneering material (*p* < 0.001) in the following decreasing order: TiO_2_, CoCrMo, ZrO_2_, and PEEK.

## 4. Discussion

The research interest in color measurement of teeth and dental restorations using different devices is increasing, especially when it comes to new materials. This includes different validation and comparative aspects, and the determination of thresholds and color interactions of human teeth and dental materials [12]. This is, to the best of our knowledge, the first study that assesses the influence of PEEK as a core material. PEEK is frequently the focus of oral rehabilitation studies, since PEEK-based materials are applied in addition to other polymers like poly(methyl methacrylate) (PMMA)-based and composite resin materials in removable and fixed partial denture technology. PEEK has become an alternative to conventional and well-investigated veneering and denture base resin materials, with low discoloration rates and improved mechanical properties [13,14,15].

Based on the results obtained, the hypotheses set as the premises of this study had to be rejected. In summary, the present study showed that the EasyShade method was not able to detect the delivered shade of A3 (A2 for VITA Mark II, A3.5 for VITA Enamic, B2 for LAVA Ultimate, and B3 for IPS e.max CAD). Accordingly, LabE-values also showed significant differences between the tested veneering materials of the same color (*p* < 0.001). Previous findings showed poor pair-agreement rates of shade matching instruments among the tested instruments (including the VITA EasyShade) ranging from 37.7% to 48.2%, and the incidence of identical shade results shared by all instruments under investigation was only 25.9% [16]. Different CieLab-System values and shade matching results were reported for identical teeth; therefore, a combination of shade matching instruments and visual shade confirmation was recommended for clinical use. In general, dental spectrophotometers rarely exhibit comparable shade selection outputs [16,17].

In the present study, however, both measurement methods, *i.e.*, the VITA EasyShade and the CieLab-System, showed that the core (*p* < 0.001) and veneering material (*p* < 0.001), as well as the thickness of the veneering material (*p* < 0.001) of different assemblies, have a significant impact, which elucidates an inherent color difference in the different assemblies. These findings are not surprising, as it has been shown that especially metal substructures and different porcelains affect the final color of restorations [18]. Moreover, the porcelain thickness has been found to have an impact on chroma [19,20].

The overall appearance and perception of dental restorations depends, however, on several factors: the color of the adjacent teeth, light scattering effects, and inherent material characteristics such as opacity and translucency. Detectable color differences to the human eye are normally non-discernible below Δ*E*-values of 1, which change into an unacceptable color set at Δ*E* when more than 3.3 [21,22,23]. In this light, the obtained values may be of clinical significance.

As a first general shortcoming of the present investigation, it must be pointed out that the base and veneering specimens were not superimposed with adhesion, *i.e.*, no melting fusion or bonding/luting procedures were performed. This leaves the possibility of light scattering between the different samples. Another potentially critical factor in the methodology is that the specimens were polished, mainly aiming to reduce any additional scattering effects. However, this does not represent the realistic surface characteristics of routinely machined and milled specimens when using CAD/CAM devices. Finally, neither opaque luting materials nor colored cements were used, which has been shown to positively influence the optical behavior of CAD/CAM glass-ceramic lithium disilicate-reinforced restorations [24]. Therefore, our results should be interpreted with caution as the set-up probably led to more accentuated color differences. In favor of the present set-up, however, one may argue that it may be regarded as a worst-case scenario, depicting differences in core and veneering materials for screening purposes of a new core material, *i.e.*, PEEK.

In the present study, four different restorative materials were used. They represent frequently used ceramic and polymer materials in CAD/CAM technology. In general, some variation in terms of translucent and fluorescent properties when compared with glass-ceramics of the same color was found [25]. In addition, it was shown that full-ceramic systems rarely match the color of the shade guide, which was also corroborated by the present study [26]. Specimens made from semi-translucent all-ceramic systems exhibited clinical shade matches that were superior to those made with the metal ceramic systems. Increasing thickness of the semi-translucent systems from 1.0 to 2.0 mm did not improve shade matching [27].

## 5. Conclusions

Within the limitations of the present study, the following conclusions can be drawn:

- PEEK as core material showed no differing tendencies when compared to gold standard core materials such as ZrO_2_ and CoCrMo with respect to the CieLab-System parameters of the assemblies and the modification of the CieLab-System parameters for each individual veneering material.
- Different veneering materials showed different CieLab-System and VITA EasyShade outcomes.
- The relative frequency of the measured VITA EasyShade parameters of PEEK cores showed comparable results with the gold standard CoCrMo, regardless of the veneering material used.
- Core materials and the modification between assembly and veneering material showed significant impact on the CieLab-System and VITA Easy Shade results, *i.e.*, the combination of core and veneering material was influential.
- Veneering materials influenced the VITA EasyShade parameters of the combination from core and veneering.

## Figures and Tables

**Table 1:** Overview of the materials tested in this study.

Brands	Material Type	Manufacturer	Batch No.	Composition
**Core Materials**				
Dentokeep	PEEK	nt-trading, Karlsruhe, Germany	11DK14Q01	PEEK, 20 wt % anorganic fillers
IPS e.max ZirCAD	ZrO_2_	Ivoclar Vivadent, Schaan, Liechtenstein	N35700	ZrO_2_, HFO_2_, Al_2_O_3_ and other oxides
Remanium GM 800+	CoCrMo	Dentanium, Ispringen, Germany	816	Co 63.3 wt %, Cr 30 wt %, Mo 5 wt %
Bio-Titan	TiO_2_	DCS Dental AG, Allschwil, Switzerland	8797	pure titanium grade 4
**Veneering Materials**				
VITA Mark II A3	glass-ceramic	VITA Zahnfabrik, Bad Säckingen, Germany	29380	SiO_2_: 56–64 wt %, Al_2_O_3_: 20–23 wt %, Na_2_O: 6–9 wt %, K_2_O: 6–8 wt %, CaO: 0.3–0.6 wt %
IPS e.max CAD A3	lithium disilicate glass-ceramic	Ivoclar Vivadent, Schaan, Liechtenstein	S14448	SiO_2_, Li_2_O, K_2_O, MgO, Al_2_O_3_, P_2_O_5_ and other oxides
LAVA Ultimate	resin nano ceramic	3M ESPE, Seefeld, Germany	N435300	Polymer with appr. 80 wt % anorganic filler
VITA Enamic	hybrid dental ceramic	VITA Zahnfabrik, Bad Säckingen, Germany	36810	86 wt % feldspar ceramic, 14 wt % polymer

**Table 2:** Descriptive statistics of the CieLab-System parameters (mean, standard deviation (SD) of veneering materials together with VITA EasyShade evaluations.

VITA EasyShade	Lab-Values	VITA Mark II	VITA Enamic	LAVA Ultimate	IPS e.max CAD
		A2	A3.5	B2	B3
CieLab-System	*L* mean (SD)	62.7 (1.3) ^b^	60.4 (1.3) ^a^	60.1 (1.9) ^a^	62.7 (0.7) ^b^
	*a* mean (SD)	−0.1 (0.6) ^c^	0.9 (0.7) ^d^	−1.6 (0.4) ^a^	−0.5 (0.8) ^b^
	*b* mean (SD)	9.0 (2.9) ^b^	11.5 (2.4) ^c^	2.4 (2.3) ^a^	9.7 (2.4) ^b^
	*E* mean (SD)	63.4 (1.5) ^c^	61.5 (1.7) ^b^	60.2 (1.9) ^a^	63.6 (0.9) ^b^

^a**,**b**,**c**,**d^ different letters show significant differences between tested veneering materials within one CieLab-System parameter.

**Table 3:** Relative frequency (%) and 95% CI of the measured VITA EasyShade parameters of all assemblies between core and veneering material, regardless of the veneering thickness and core materials (left)/veneering materials (right).

**Veneering Materials**				
**Tooth Shade (Chairside)**	**VITA Mark II (A2)**	**VITA Enamic (A3.5)**	**LAVA Ultimate (B2)**	**IPS e.max CAD (B3)**
A1	39.4 (32;47)	0 (0;2)	36.9 (30;45)	14.9 (7.6;29.6)
A2	36.3 (29;44)	10.6 (7;16)	18.8 (13;26)	12.5 (8;19)
A3	15.6 (11;22)	12.5 (8;19)	3.8 (2;8)	10.1 (4.8;14.0)
A3.5	0 (0;2)	13.1 (9;19)	0 (0;2)	0 (0;2)
A4	0 (0;2)	8.1 (5;13)	0 (0;2)	0 (0;2)
B1	0.6 (0;3)	0 (0;2)	5.3 (3;10)	0 (0;2)
B2	8.1 (5;13)	1.9 (1;5)	31.9 (25;39)	20.6 (15;28)
B3	0 (0;2)	53.8 (46;61)	0 (0;2)	46.3 (39;54)
C2	0 (0;2)	0 (0;2)	3.8 (2;8)	0 (0;2)
**Core Materials**				
**Tooth shade (chairside)**	**PEEK**	**ZrO_2_**	**CoCrMo**	**TiO_2_**
A1	25 (19;32)	25.6 (19;33)	25.6 (19;33)	6.9 (4;12)
A2	16.9 (12;23)	9.4 (6;15)	16.3 (11;23)	35.6 (29;43)
A3	17.5 (12;23)	18.8 (13;26)	10 (6;16)	0 (0;2)
A3.5	0 (0;2)	3.1 (3;10)	0.6 (0;3)	9.4 (6;15)
A4	0 (0;2)	0 (0;2)	0 (0;2)	8.1 (5;13)
B1	0 (0;2)	5.6 (3;10)	0 (0;2)	0 (0;2)
B2	9.4 (6;15)	19.4 (14;25)	17.5 (12;24)	16.3 (11;23)
B3	31.3 (25;39)	18.1 (13;25)	30.6 (24;38)	20 (15;27)
C2	0 (0;2)	0 (0;2)	0 (0;2)	3.8 (2;8)

**Table 4:** Descriptive statistics of modifications of the CieLab-System parameters (baseline: veneering material) of all tested material assemblies, regardless of the veneering thickness.

VITA EasyShade	Lab-Values	VITA Mark II/A2	VITA Enamic/A3.5	LAVA Ultimate/B2	IPS e.max CAD/B3
PEEK					
CieLab-System	∆*L* mean (SD)	−12.1 (5.2)	−11.2 (5.9)	−13.4 (6.6)	−10.3 (5.2)
	∆*a* mean (SD)	−0.8 (0.3)	−1.4 (0.1)	−2.3 (0.5)	−1.9 (0.1)
	∆*b* mean (SD)	−7.4 (1.8)	−7.6 (1.6)	−11.7 (1.5^)^	−8.4 (2.1)
	∆*E* mean (SD)	14.3 (5.1)	13.8 (5.8)	18.4 (5.4)	13.6 (5.3)
ZrO_2_					
CieLab-System	∆*L* mean (SD)	−10.5 (4.8)	−10.1 (5.4)	−12.1 (6.1)	−9.2 (4.7)
	∆*a* mean (SD)	−1.1 (0.5)	−0.5 (0.3)	−1.1 (0.1)	−1.1 (0.1)
	∆*b* mean (SD)	−5.4 (1.6)	−6.0 (0.9)	−8.4 (1.5)	−6.8 (1.5)
	∆*E* mean (SD)	12.1 (4.6)	11.9 (5.0)	15.0 (5.8)	11.7 (4.6)
CoCrMo					
CieLab-System	∆*L* mean (SD)	−10.4 (4.7)	−9.8 (5.4)	−11.7 (6.1)	−8.9 (4.6)
	∆*a* mean (SD)	−0.3 (0.3)	−0.8 (0.1)	−1.4 (0.1)	−1.3 (0.1)
	∆*b* mean (SD)	−5.5 (1.6)	−5.9 (1.1)	−8.3 (1.8)	−6.7 (1.6)
	∆*E* mean (SD)	11.9 (4.5)	11.6 (5.1)	14.5 (6.0)	11.3 (4.6)
TiO_2_					
CieLab-System	∆*L* mean (SD)	−4.8 (2.3)	−4.5 (2.5)	−5.3 (2.9)	−4.1 (2.1)
	∆*a* mean (SD)	−0.5 (0.5)	−0.7 (0.4)	−1.6 (0.6)	−1.1 (0.5)
	∆*b* mean (SD)	−4.2 (1.8)	−4.1 (1.6)	−5.3 (2.2)	−4.0 (1.6)
	∆*E* mean (SD)	6.5 (2.9)	6.2 (2.9)	7.7 (3.6)	5.8 (2.7)

## Abbreviations

The following abbreviations are used in this manuscript:

ZrO_2_	zirconia
TiO_2_	titanium oxide
CoCrMo	cobalt–chromium–molybdenum alloy
CAD/CAM	computer-aided design/computer-aided manufacturing
